# Diminished Medial Prefrontal Activity behind Autistic Social Judgments of Incongruent Information

**DOI:** 10.1371/journal.pone.0039561

**Published:** 2012-06-22

**Authors:** Takamitsu Watanabe, Noriaki Yahata, Osamu Abe, Hitoshi Kuwabara, Hideyuki Inoue, Yosuke Takano, Norichika Iwashiro, Tatsunobu Natsubori, Yuta Aoki, Hidemasa Takao, Hiroki Sasaki, Wataru Gonoi, Mizuho Murakami, Masaki Katsura, Akira Kunimatsu, Yuki Kawakubo, Hideo Matsuzaki, Kenji J. Tsuchiya, Nobumasa Kato, Yukiko Kano, Yasushi Miyashita, Kiyoto Kasai, Hidenori Yamasue

**Affiliations:** 1 Department of Physiology, The University of Tokyo, Bunkyo-ku, Tokyo, Japan; 2 Department of Neuropsychiatry, The University of Tokyo, Bunkyo-ku, Tokyo, Japan; 3 Department of Child Neuropsychiatry, The University of Tokyo, Bunkyo-ku, Tokyo, Japan; 4 Department of Radiology, Graduate School of Medicine, The University of Tokyo, Bunkyo-ku, Tokyo, Japan; 5 Global Center of Excellence (COE) Program, The University of Tokyo, Bunkyo-ku, Tokyo, Japan; 6 Department of Radiology, Nihon University School of Medicine, Itabashi-ku, Tokyo, Japan; 7 Japan Science and Technology Agency, CREST, Chiyoda-ku, Tokyo, Japan; 8 Osaka-Hamamatsu Joint Research Center for Child Mental Development, Hamamatsu University School of Medicine, Hamamatsu, Japan; 9 Department of Neuropsychiatry, Showa University School of Medicine, Tokyo, Japan; Yale University, United States of America

## Abstract

Individuals with autism spectrum disorders (ASD) tend to make inadequate social judgments, particularly when the nonverbal and verbal emotional expressions of other people are incongruent. Although previous behavioral studies have suggested that ASD individuals have difficulty in using nonverbal cues when presented with incongruent verbal-nonverbal information, the neural mechanisms underlying this symptom of ASD remain unclear. In the present functional magnetic resonance imaging study, we compared brain activity in 15 non-medicated adult males with high-functioning ASD to that of 17 age-, parental-background-, socioeconomic-, and intelligence-quotient-matched typically-developed (TD) male participants. Brain activity was measured while each participant made friend or foe judgments of realistic movies in which professional actors spoke with conflicting nonverbal facial expressions and voice prosody. We found that the ASD group made significantly less judgments primarily based on the nonverbal information than the TD group, and they exhibited significantly less brain activity in the right inferior frontal gyrus, bilateral anterior insula, anterior cingulate cortex/ventral medial prefrontal cortex (ACC/vmPFC), and dorsal medial prefrontal cortex (dmPFC) than the TD group. Among these five regions, the ACC/vmPFC and dmPFC were most involved in nonverbal-information-biased judgments in the TD group. Furthermore, the degree of decrease of the brain activity in these two brain regions predicted the severity of autistic communication deficits. The findings indicate that diminished activity in the ACC/vmPFC and dmPFC underlies the impaired abilities of individuals with ASD to use nonverbal content when making judgments regarding other people based on incongruent social information.

## Introduction

Individuals with autism spectrum disorders (ASD) have difficulties in processing of both verbal and nonverbal information [1]–[8]. Previous studies have demonstrated that they frequently underutilize verbally-supplied knowledge about other people, such as regarding their backgrounds and gender [5], [9], [10]. Other studies have revealed that they often inappropriately process nonverbal social information including facial expressions, voice prosody, and eye gaze [2], [3], [6], [11]–[14]. Consequently, individuals with ASD often experience difficulty in interpreting complex social information, particularly when the verbal and nonverbal contents conflict with one another, which partially causes their frequent misinterpretation of irony and humor in real-life situations [9], [15], [16].

The characteristic behavioral responses to incongruent verbal-nonverbal information of ASD individuals have been reported in a previous study [17]. Compared with typically developed (TD) individuals, ASD individuals showed less sensitivity to the nonverbal content in incongruent information, even when they could effectively evaluate the verbal and nonverbal contents in isolation. This report is consistent with other behavioral studies that demonstrated that there are greater differences in the ability to process nonverbal information between ASD and TD individuals than in the ability to process verbal information [18], . Although these observations imply that there are specific neural deficits that underlie this characteristic behavior in ASD individuals, to the best of our knowledge, no previous study has directly examined the neural deficits in ASD individuals that mediate their reduced use of nonverbal content when processing incongruent verbal-nonverbal social information.

Autistic responses to other types of incongruity have been investigated in a series of previous studies [4], [5], [7], [20], [21]. Previous neuroimaging studies revealed a neural mechanism underlying autistic responses to incongruity between *a priori* language-based knowledge and novel language-based information [5], [7], [21]. One of the studies revealed that the ventromedial prefrontal cortex in ASD individuals, compared with that in TD, did not show significant responses to pragmatic incongruity [5]. Another study demonstrated that activity in the left inferior frontal cortex is reduced in people with ASD during the integration of semantic social information [7]. Other neuroimaging studies examined neural response in individuals with ASD to incongruity between different types of nonverbal information (e.g., facial expression vs. voice prosody) [20] or their responses to stories provided as several sentences [4]. A study using cartoons revealed that the medial prefrontal and superior temporal regions in ASD subjects showed decreased activity during the presentation of ironic content [20]. Despite these findings on the neural basis of social incongruity processing in ASD individuals, little is known about the neural mechanisms underlying the characteristic autistic behavioral response to incongruity between verbal and nonverbal information.

**Figure 1:**
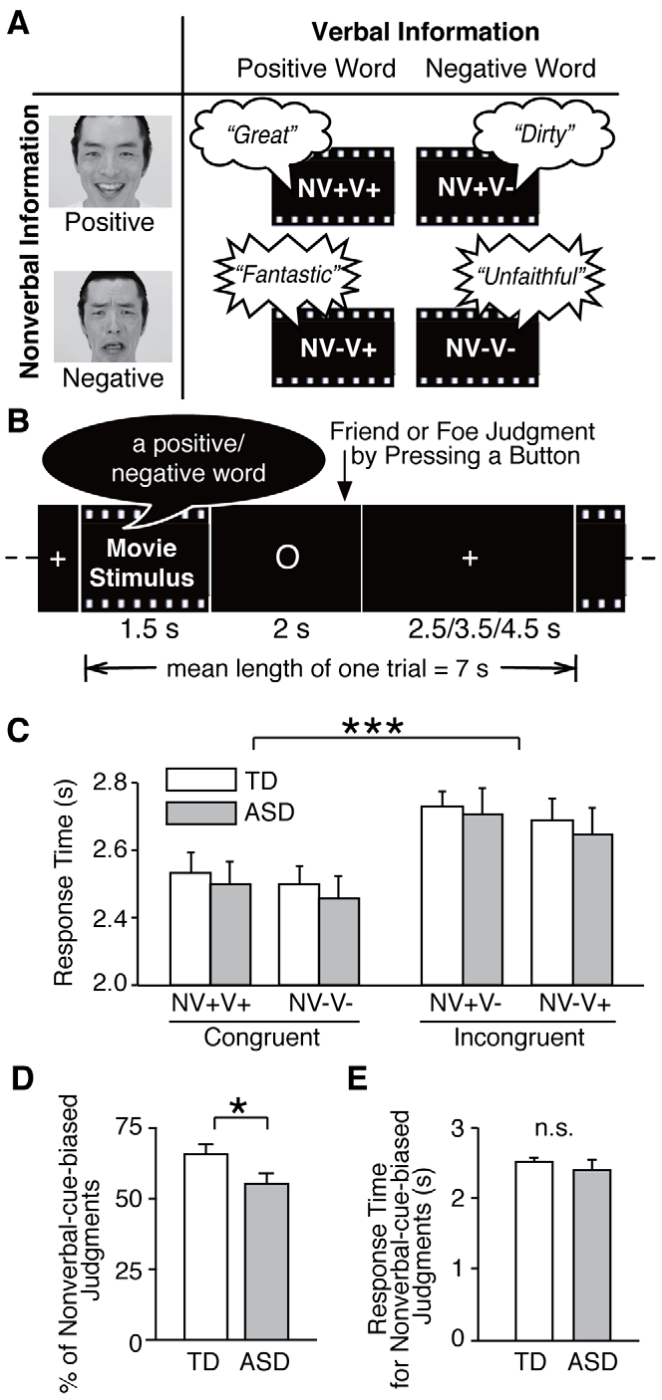
Task design and behavioral results. **(A) Short movie stimuli.** Our stimuli were short movies in which different professional actors spoke different emotional words (verbal information; positive and negative) while displaying different emotional facial expressions and voice prosody (nonverbal information; positive and negative). The emotional valence of the facial expressions was always the same as that of the voice prosody. Overall, the stimuli consisted of two types of congruent short movies (NV+V+: positive nonverbal and positive verbal information; NV-V-: negative nonverbal and negative verbal information) and two types of incongruent ones (NV+V-: positive nonverbal and negative verbal information; NV-V+: negative nonverbal and positive verbal information). **(B) Task design.** One trial of fMRI scanning session consisted of a 1.5 sec movie stimulus period, 2 sec response period, and a 2.5,3.5, or 4.5 sec fixation period. Participants were instructed to judge the person in each movie as friend or foe by pressing the corresponding buttons. **(C) Response times.** In a repeated-measure mixed-design two-way ANOVA, there was no significant main effect of group (TD versus ASD) on response time, whereas there was a significant main effect of stimulus type (congruent versus incongruent). Response times for the incongruent stimuli were significantly longer than that for the congruent stimuli (***: P<0.001). To present the data, we further divided the stimulus types into more detailed categories. **(D) Number of nonverbal-cue-biased judgments.** ASD individuals exhibited significantly fewer nonverbal-cue-biased judgments of incongruent stimuli than the TD individuals (*:P<0.05) **(E) Response times for nonverbal-cue-biased judgments.** There was no significant difference in response time for nonverbal-cue-biased judgments of incongruent stimuli between the ASD and TD groups.

In the present functional magnetic resonance imaging (fMRI) study, therefore, we aimed to reveal the neural mechanism underlying the imbalance between the interpretation of incongruent verbal-nonverbal social information in ASD individuals using realistic movie stimuli. In each of the short movie stimuli, a different professional actor spoke a word that was related to a positive or negative emotion while producing a positive or negative emotion-related facial expression and voice prosody (Fig. 1A). To directly investigate the neural response to this verbal-nonverbal incongruity, we did not give the participants any background knowledge or contextual information about the actors in the movies before conducting the fMRI scanning. Furthermore, we used a naturalistic psychological task wherein the participants responded as spontaneously as they do in real-life situations, because a previous behavioral study showed that autistic behavioral patterns are likely to be expressed in such spontaneous and automatic responses to social stimuli [19]. In the present study, the participants were instructed to judge an actor shown in a movie as a friend or a foe without explicit instruction about the incongruity itself, because the processing of incongruent social information occurs automatically in real-life situations, even when the subject does not explicitly attend to the incongruity (i.e., the Stroop effect has negligible influence) [22]. We analyzed the data according to the type of information that primarily affected the friend or foe judgments, and the judgments of the incongruent stimuli were classified into nonverbal-cue-biased or verbal-cue-biased judgments.

Based on previous behavioral findings [17]–[19], we hypothesized that the participants with high-functioning ASD would place disproportionately less emphasis on nonverbal content and make less frequent nonverbal-cue-biased judgments than TD participants. Hence, we assumed that the most characteristic impaired neural response in ASD individuals would be a difference in brain activity between verbal- and nonverbal-cue-biased judgments. To test this hypothesis, we compared the brain activity during nonverbal-cue-biased judgments in the ASD group with that in the TD group.

To focus on neural deficits mediating impaired social judgments in ASD individuals, we searched brain areas that are considered to be related to social judgments. Specifically, we estimated brain activity in anatomically-defined brain regions consisting of the medial prefrontal cortex (mPFC), precuneus, temporoparietal junction (TPJ), amygdala, superior temporal sulcus (STS), anterior insula (AI) and inferior frontal gyrus (IFG). According to several previous studies (see reviews in [23]–[25]), these regions are considered to underlie empathy processing, the mentalizing system, and uncertainty judgments, which are thought to be involved in social judgments.

## Materials and Methods

### Ethics Statement

The experiment protocol used in this study was approved by the ethics committee of The University of Tokyo Hospital (#1350). After a complete explanation of the study, written informed consent was obtained from all participants. Using longitudinal clinical assessments, a trained psychiatrist (H.Y.) confirmed that all of these adult participants had no intellectual disabilities, no need for psychotropic medication, and were capable of providing informed consent. Therefore, no participant needed someone else who consented on the behalf of the participants.

### Participants

Fifteen male participants with ASD were recruited from the outpatient service of The University of Tokyo Hospital (Table 1). Fourteen were clinically diagnosed with high-functioning autism, while the remaining subject was diagnosed with pervasive developmental disorder-not otherwise specified. These diagnoses were based on the strict criteria of the Diagnostic and Statistical Manual-Revision IV [26], and they were conducted in consensus by two trained child-adolescent psychiatrists with more than ten years of clinical experience (H.Y. and N.K.) following more than two months of follow-up examinations. Another child adolescent psychiatrist (H.K.) confirmed the diagnoses of some patients using the Japanese version of the Autism Diagnostic Interview-Revised (ADI-R) [27]. For all participants who did not meet the threshold in the ADI-R social domain, the group was confirmed by the Childhood Autism Rating Scale (CARS) [28]. Previous studies have suggested that individuals classified as autistic according to both ADI-R and CARS had significantly lower IQ than those classified by CARS alone [29]. It is reasonable to suggest that the subsample of high-functioning ASD participants who did not meet ASD criteria based on ADIR, which is rated based on descriptive information by caregivers, could be classified with ASD based on CARS, which is rated by clinicians based on direct observations on behavior [30]. Thirteen of the 15 participants had never been prescribed psychotropic medication, whereas the other two has not taken medication for at least the previous seven months. The participants completed verbal and performance IQ tests, and they all exhibited estimated IQs ranging from average to above average. Seventeen healthy male TD participants were also recruited. All of the ASD and TD participants were interviewed by a trained psychiatrist (H.Y.) to screen for the presence of neuropsychiatric disorders using the Structured Clinical Interview for DSM-IV Axis I Disorder [31]. There were no significant differences in mean age, parental socioeconomic status (SES), handedness, or IQ between the ASD and TD groups.

**Table 1:** Demographic characteristics of the participants.

Variables	Subjects with ASD	TD controls	t value (p)
	(n = 15)	(n = 17)	
Age (Range) (SD)	28.2 (20–44) (7.4)	30.3 (21–41) (5.7)	0.906 (.372)
Height, cm (SD)	170.8 (3.9)	174.3 (6.5)	1.8 (.080)
Body weight, kg (SD)	64.1 (9.4)	70.5 (9.8)	1.9 (.068)
SES* (SD)	3.3 (1.0)	1.8 (0.4)	5.5 (<0.001)
Parental SES* (SD)	2.1 (0.5)	2.1 (0.6)	0.2 (.817)
Handedness: Right/Mixed/Left	14/1/0	17/0/0	
IQ**			
FIQ (SD)	104.6 (11.0)	109.2 (7.2)	1.4 (.166)
VIQ (SD)	111.8 (15.3)		
PIQ (SD)	90.0 (14.0)		
Autism spectrum disorder subtype	14 HFA/1 PDD-NOS***		
Autism Diagnostic Interview-Revised			
Social (SD)	13.4 (6.3)		
Communication (SD)	12.1 (3.5)		
Repetitive (SD)	4.6 (2.4)		
Autism Spectrum Quotient (SD)	37.4 (7.1)	13.9 (4.9)	10.3 (<0.001)

* Socioeconomic status assessed using the Hollingshead. Higher scores indicate lower status.

** The intelligence quotients were measured using the Wechsler Adult Intelligence Scale in ASD participants and a Japanese version of the National Adult Reading Test in the TD participants.

*** HFA: High-functioning autism; PDD-NOS: Pervasive developmental disorder-not otherwise specified.

The exclusion criteria for the both groups consisted of any history of current or past neurological illness, traumatic brain injury with any known cognitive consequences or loss of consciousness for more than five minutes, and substance abuse or addiction. An additional exclusion criterion for the TD group was a history of psychiatric disease or a family history of an axis I disorder among their first-degree relatives.

### Details of the Questionnaires

Handedness was determined using the Edinburgh Handedness Inventory [32], and the participants with a laterality index of more than 0.5 were regarded as right-handed. The participants whose laterality index score ranged from –0.5 to 0.5 were defined as ambidextrous. The SES of the participants and their parents was assessed using the Hollingshead scale [33]. All participants completed a Japanese translation [34] of the 50-item Autism-Spectrum Quotient [35] within one month before or after the fMRI scanning. The IQ of the TD group was estimated using a Japanese version [36] of the National Adult Reading Test (NART) [37]. Although the NART can measure IQ accurately in TD participants, the test is problematic for ASD participants because of the well-known imbalances in their intellectual abilities. Therefore, the IQs of the ASD participants were assessed using the full scale of the Wechsler Adult Intelligence Scale Revised Japanese version [38]. Sleepiness during the MR scans was assessed using the Stanford Sleepiness Scale [39] and no participants fell asleep during the fMRI experiment.

### MRI Scanning

A 3T MRI scanner (GE) was used in this experiment. The anatomical scanning sequence was a three-dimensional Fourier-transform spoiled-gradient-recalled acquisition during steady state (TR = 6.8 s, slice thickness = 1 mm, in-plane resolution = 1 × 1 mm). Gradient-echo echo-planar sequences were used for functional imaging (TR = 3 s, TE = 35 ms, flip angle = 80 degree, cubic voxel of 4 mm, 22 slices, from the ventral to dorsal slices). The first five functional images in each run were excluded from analysis to account for the equilibrium of longitudinal magnetization. Because the stimuli included human speech, we used an MRI-compatible headphone system (Hitachi, Corp. Tokyo, Japan), and confirmed before and after the experiment that the speech content and prosody in the stimuli could be heard clearly. A trained neuroradiologist (H.T. or O.A.) evaluated the MRI scans and found no gross abnormalities in any participant. Magnetic field inhomogeneities in our scanner were monitored with basic quality control conducted daily, and they were stable over the course of this study.

### Task and Stimuli

The stimuli were 80 original monochrome movies with a length of 1,500 ms (see Supplementary materials). In each movie, one of 20 professional actors (10 male and 10 female) spoke a different emotional word accompanied by an emotional facial expression and expressive prosody. To exclude unnatural and unrealistic stimuli, healthy Japanese adults (T.W., N.Y., Y.K., and H.Y.) examined whether the movies were natural and realistic or not. Only the movies that were rated as the most realistic and natural were used as the stimuli in the present experiment.

As verbal information, words with high emotional valence and arousal were selected from the list of Affective Norms for English Words (40 words had a positive valence, whereas the other 40 had a negative valence) [40]. Overly aggressive words were excluded for ethics considerations. As nonverbal information, the professional actors were instructed to display happy or disgusted facial expressions and prosody while speaking each word. The emotional directionality of the facial expressions was always the same as that of the accompanying voice prosody. Thus, there were four types of stimuli (Fig. 1A): a disgusted facial expression and prosody paired with a negative word (i.e., negative (-) nonverbal (NV) information and negative (-) verbal (V) information; NV-V-, e.g., Video S1), a disgusted facial expression and prosody paired with a positive word (NV-V+, e.g., Video S2), a happy facial expression and prosody paired with a negative word (NV+V-, e.g., Video S3), and a happy facial expression and prosody stimuli with a happy word (NV+V+, e.g., Video S4). There were 20 videos of each type.

The emotional directionality of the nonverbal information was validated in an independent behavioral experiment using a separate group of 12 TD male participants (24–34 years old) who did not undergo the subsequent fMRI experiments. To validate the nonverbal information conveyed by the actors’ facial expressions, the participants were instructed to evaluate silent movies by indicating a score on an 8-point happy–disgust scale, with one being the happiest and 8 being the most disgusted. In contrast, to validate the prosody, we had the participants perform the same evaluation of the voice stimuli without the visual component of the movie (i.e., only listening to voice). In these evaluations of voice prosody, the participants were instructed to ignore the verbal content of the speech as much as possible. The mean scores and standard deviations for the facial expressions were 1.9±0.3 in the NV+V-, 1.8±0.2 in the NV+V+, 7.1±0.6 in the NV-V-, and 6.9±0.5 in the NV-V+ conditions. The mean scores and standard deviations for the voice prosody 3.0±0.3 in the NV+V-, 2.7±0.4 in the NV+V+, 6.8±0.5 in the NV-V-, and 6.6±0.4 in the NV-V+ conditions. There was no main effect of verbal information in a repeated-measures analysis of variance (ANOVA) comparing the positive versus negative words. All scores were significantly different from neutral facial expressions or prosody (P = 0.002, one-sample t-tests). These results indicate that the intended nonverbal information was appropriately conveyed to the participants, for both the facial expressions and voice prosody.

The fMRI scanning consisted of two runs, each of which involved 10 NV-V-, 10 NV-V+, 10 NV+V-, and 10 NV+V+ stimuli, pseudo-randomly ordered. During scanning, the participants were presented with the movies sequentially, and were instructed to judge whether they believed the actor in the movie was a friend or a foe by pressing a button. They were asked to make an intuitive judgment of the actors without deep consideration. Each movie stimulus was presented immediately before a 2,000-ms response period, followed by a 2,500–4,500-ms waiting period (Fig. 1B). The mean length of each trial across all 80 trials was 7,000 ms. For optimization of the event-related design, we also added 10 7,000 ms dummy trials in each run. Therefore, each run took approximately six minutes. Before fMRI scanning, all participants underwent sufficient training to allow them to complete the judgment task.

#### Behavioral Analysis

Before the following analysis, we needed to exclude the possibility that the friend or foe judgments were strongly biased by any exogenous feature in the stimuli. If this was the case, the stimuli may have been disproportionately judged as either friends or foes no matter who saw them. To address this issue, we applied a chi-square goodness-of-fit test to the behavioral data obtained from the TD subjects, and we found that there was no significant bias towards judging the stimuli as a friend or a foe across all of the incongruent stimuli (chi-square = 15.7, degree of freedom = 16, P>0.47). This suggests that the judgments cannot be explained by the exogenous features of the stimuli rather than internal processes in the participants.

We classified the judgments of the incongruent stimuli into nonverbal-cue-biased and verbal-cue-biased judgments, according to the type of information that primarily affected the judgment [17]. For example, making a judgment that the actor was a foe in response to a NV-V+ stimulus was regarded as a nonverbal-cue-biased judgment, and making a judgment that the actor was a foe in response to a NV+V- stimulus was regarded as a verbal-cue-biased judgment. To clarify the behavioral characteristics of the ASD group, we first conducted repeated-measures mixed-design ANOVAs of response time with one within-participants factor (type of stimuli: congruent/incongruent stimuli) and one between-participants factor (group: ASD/TD). To examine the effects of conflict adaptation [41]–[43], paired t-tests were conducted between the response time for a congruent stimulus or an incongruent stimulus. We then compared the number of nonverbal-cue-biased judgments of incongruent stimuli between the TD and ASD groups using two-sample t-tests. Note that it is not necessary to compare the number of verbal-cue-biased judgments between the TD and ASD groups, because the number is always the converse of the number of the nonverbal-cue-biased judgments. The influence of type of emotion conveyed by the nonverbal information on the number and mean response time of the nonverbal-cue-biased judgments was also estimated as the interaction between the group and type of emotion conveyed in the stimulus in a repeated-measure mixed-design two-way ANOVA (group: TD/ASD × emotional type: positive/negative). Statistical significance was set at P<0.05.

#### Preprocessing of fMRI Data

The fMRI data were analyzed in SPM8 (http://www.fil.ion.ucl.ac.uk/spm/). Functional images were realigned, slice timing corrected, normalized to the default template with interpolation to a 2×2×2 mm space [44], and smoothed (full width half maximum = 8 mm, Gaussian filter). To remove low-frequency drift from the data, high-pass temporal filtering with a cut-off of 128 s was applied. There was no significant difference in the extent of motion during the scanning between the TD and ASD participants as a repeated-measure mixed-design ANOVA (TD/ASD × absolute values of the six motion parameters: x/y/z/pitch/yaw/row) found neither a significant main effect of the group or interaction (P>0.4).

### fMRI Analysis

For our event-related fMRI design, in single level analyses, we used a general linear model with eight regressors: four types of stimuli (NV-V-, NV+V-, NV+V-, and NV+V+) × two types of response (friend and foe). Each event-related regressor had an onset at the time of stimulus presentation and a duration that corresponded to the response time. We also added six regressors for the six motion correction parameters. We then defined the brain activity during judgments biased to nonverbal cues as Bnv: [“Foe to NV-V+” and “Friend to NV+V-”] > [“Foe to NV-V-” and “Friend to NV+V+”]. This contrast was based on the assumption that, in judgments of foe to NV-V+, for example, the participants emphasized nonverbal information more explicitly than in judgments of foe to NV-V-. To make this analysis valid, we excluded types of judgments that were rarely selected (e.g., “Friend to NV-V-“ and “Foe to NV+V+”). This exclusion was intended to reduce the influence of the stimuli in which the emotional content of the facial expressions and voice prosody were not exactly congruent to the verbal content of the words, though the effect cannot be completely excluded. Using the same logic, we defined the brain activity during judgments biased to verbal cues as Bv: [“Friend to NV-V+” and “Foe to NV+V-”] > [“Friend to NV+V+” and “Foe to NV-V-”].

For the group level analysis, we estimated that activity in different brain regions was specifically related to nonverbal-cues-biased judgments using differences between the Bnv and Bv conditions.

The group level analysis was based on a random-effects model, and we first conducted within-group comparison for each of the groups. Moreover, we employed paired-t-tests and compared brain activity during the nonverbal-cue-biased judgments with that during the verbal-cue-biased judgments (Bnv > Bv). We then conducted between-group comparisons by employing a repeated-measure mixed-design two-way ANOVA of the brain activities (type of judgments: nonverbal- or verbal-cue-biased judgments × group: ASD/TD). Using masked images, significant brain activations were examined in anatomically-defined regions described earlier, which previous studies have suggested are related to empathy, mentalizing, or uncertainty judgments [24], [25], [45] (P<0.05, FDR-corrected, [46], [47]). These regions were defined by an automated anatomical labeling (AAL) [48] atlas implemented in WFU PickAtlas (http://www.fmri.wfubmc.edu). To account for individual anatomical differences, we applied Gaussian smoothing (full width half maximum = 8 mm) to these mask images. To investigate the involvement of other brain regions, we also conducted a whole brain analysis (P<0.05, FDR-corrected). Furthermore, for the regions found to exhibit activity in these analyses, we examined the effects of the affective valence of the verbal and nonverbal information (i.e., positive/negative) by conducting a repeated measures ANOVA of the activity (positive and negative × verbal and nonverbal information) in each of the subject groups.

To investigate which of the detected regions were more relevant to the nonverbal-cue-biased judgments, we examined Pearson’s correlation coefficients between the brain activity in the TD group during the nonverbal-cue-biased judgments and the number of behavioral judgments. This analysis is based on the assumption that activity in brain regions with relevance for certain behaviors significantly correlated with the corresponding behavioral scores across subjects [49]–[53]. The brain activity was extracted as an averaged percent signal change in 4-mm spheres around the coordinates of interest [54]. The significance level was set at P<0.05 after Bonferroni correction among the detected regions (i.e., because five regions were found, the threshold was defined as P<0.01 = 0.05/5 regions). To test whether these correlations were less strong in the ASD group, we also calculated the correlation in the ASD group, and compared the correlation coefficients between the TD and ASD groups using Fisher’s transformation of correlation coefficients. For further validation of regions detected by the analyses, we examined correlations between brain activity in the ASD group and the ADI-R sub-scores (social and communication). ADI-R-communication scores were included because these scores include items related to social interactions using nonverbal communicative cues (e.g., “failure to compensate verbal communication through gestures (B1)” and “lack of spontaneous imitation of actions and imitative social play (B2)”). Brain activity was extracted in the same way as described above in the analysis of behavior.

Finally, to exclude the possibility that the ASD subjects may show less overall brain activity than the TD subjects, we estimated differences in the amygdala response to congruent emotional stimuli between the groups, because previous studies reported enhanced activation of the amygdala in ASD individuals [55], [56]. We employed an FDR-corrected P<0.05 for this analysis in the amygdala.

## Results

### Behavioral Results

A repeated-measure mixed-design two-way ANOVA of the response time data (stimulus type: incongruent and congruent stimuli × group: ASD and TD) revealed neither significant main effects of group nor any significant interaction between the group and stimulus type (P>0.39). However, there was a main effect of stimulus type (F(1,30) = 6.3, P = 0.01). A post-hoc paired t-test revealed that response times in both the ASD and TD individuals were longer for incongruent than congruent stimuli (P<0.001, Fig. 1C). This supports the hypothesis that both the TD and ASD participants sensed the incongruity and attempted to resolve this conflicting information, though it is hard to definitively test this hypothesis.

Furthermore, a paired t-test did not detect any significant differences between response times to incongruent stimuli that were presented immediately following congruent stimuli compared to response times to incongruent stimuli that were presented immediately following incongruent stimuli. This finding allowed us to exclude conflict-adaptation effects in the following analysis [41]–[43].

A two-sample t-test of the number of judgments of the incongruent stimuli indicated that the ASD subjects made significantly fewer nonverbal-cue-biased judgments than the TD subjects (ASD: 23.2±1.8, TD: 26.1±1.2, mean ± s.e.m.; P<0.05, Fig. 1D). Within the nonverbal-cue-biased judgments, a repeated-measure mixed-design ANOVA of the number of the nonverbal-cue-biased judgments did not detect a significant interaction between the group (TD and ASD) and the emotional valence of the nonverbal cues (positive and negative) (P>0.4). These results suggest that the decrease in nonverbal-cue-biased judgments in the ASD subjects were commonly observed for both emotional valences of nonverbal information. The response time in the nonverbal-cue-biased judgments was not different between the ASD and TD groups (Fig. 1E). A repeated-measure ANOVA of the response times during the nonverbal-cue-biased judgments did not detect a significant interaction between the group and the emotional valence of the nonverbal information (P>0.5).

As a whole, these behavioral results suggest that, compared with the TD subjects, the ASD subjects showed altered and less frequent use of nonverbal content in incongruent verbal-nonverbal social information, which is consistent with previous behavioral findings [17].

### fMRI Results

#### Within-group analysis

**Figure 2:**
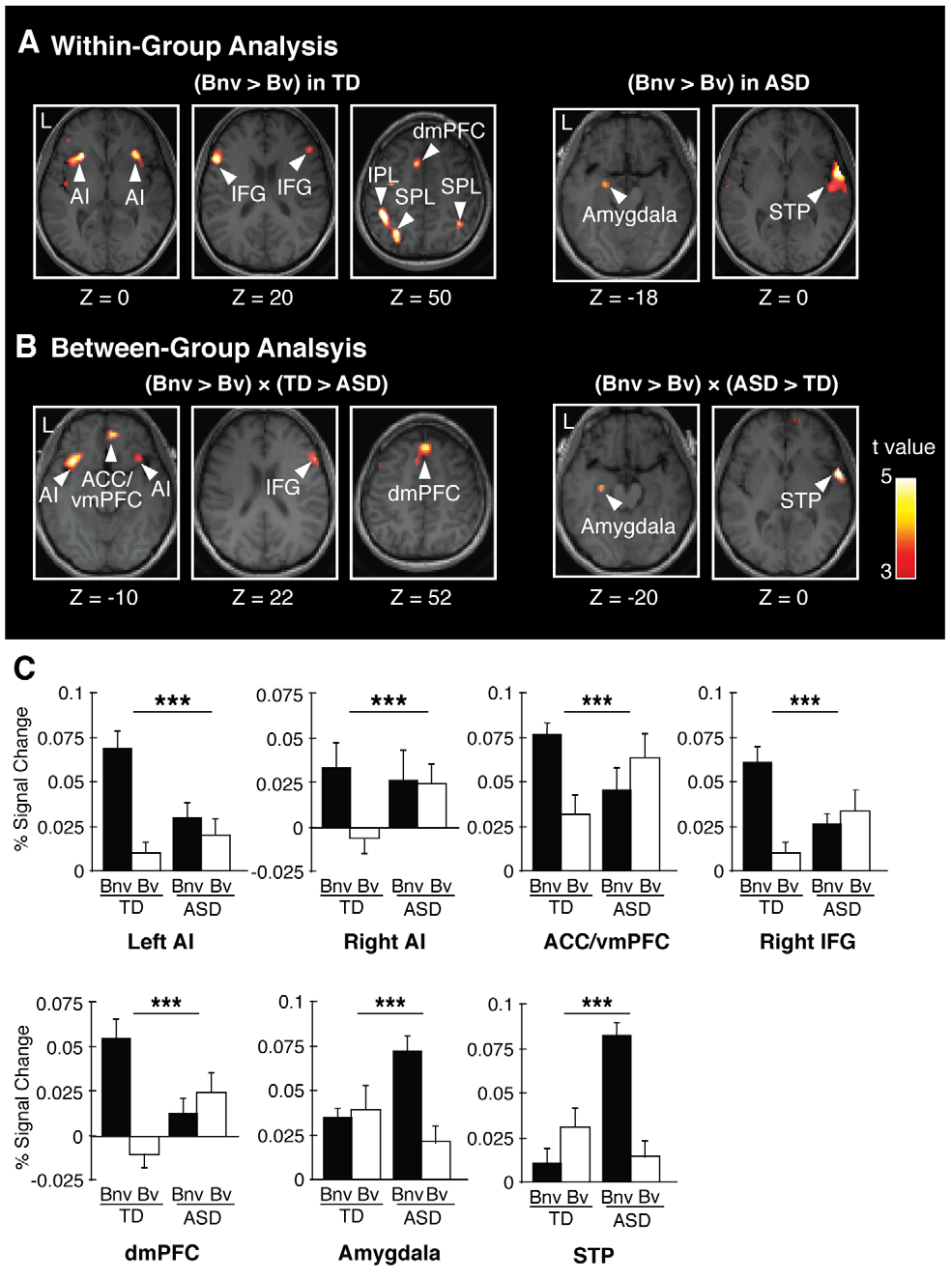
fMRI results (A) Brain regions specific to nonverbal-cue-biased judgments in TD or ASD individuals. The left three panels show the brain regions in which activity was significantly greater during the nonverbal-cue-biased judgments (Bnv) than during the verbal-cue-biased judgments (Bv) in the TD individuals (P<0.05, FDR-corrected). AI: anterior insula, IFG: inferior frontal gyrus, dmPFC: dorsal medial prefrontal cortex, IPL: inferior parietal lobe, SPL: superior parietal lobe. The right two panels show the three brain regions that had significantly greater activity during the nonverbal-cue-biased judgments than during the verbal-cue-biased judgments in the ASD individuals (P<0.05, FDR-corrected). STP: superior temporal pole. **(B) Brain regions whose activity was diminished or increased in ASD individuals.** The left three panels show the five brain regions that had a significant interaction between the type of judgment and the group, as defined as (Bnv > Bv) × (TD > ASD) (P<0.05, FDR-corrected). That is, in these regions, brain activity specific to the nonverbal-cue-biased judgments was larger in the TD group than in the ASD group. ACC/vmPFC: anterior cingulate cortex/ventral medial prefrontal cortex. The right two panels show the three brain regions in which brain activity specific to the nonverbal-cue-biased judgments was larger in the ASD group than in the TD group (P<0.05, FDR-corrected). **(C) Brain activity in the regions showing a significant interaction between judgment and group.** The bar graphs show the percent signal changes in the brain regions that were shown in panel (B). ***: P<0.001. error bar: s.d.

Before comparing the neural mechanism for the use of the nonverbal content between the ASD and TD groups, we first conducted a within-group analysis, and searched for the brain regions that showed greater activity in the nonverbal-cue-biased judgments than in the verbal-cue-biased judgments for each of the groups. In the TD group, the bilateral AI, bilateral IFG, dorsal mPFC, bilateral superior parietal lobe (SPL), and left inferior parietal lobe (IPL) showed significantly greater activity in the nonverbal-cue-biased judgments (t(16) >3.5, P<0.05, FDR-corrected; Fig. 2A; Table 2). In the ASD group, the left amygdala and right superior temporal pole (STP) exhibited significantly stronger activity specific to the nonverbal-cue-biased judgments (t(16) >3.6, P<0.05, FDR-corrected; Fig. 2A; Table 2). Furthermore, in both the TD and ASD groups, the emotional valences of the nonverbal and verbal cues did not influence the activity in these brain regions during nonverbal-cue-biased judgments (main effect of the emotional valence: P>0.3 in one-way ANOVA). These findings provide a qualitative suggestion that the ASD participants used a different neural mechanism to process nonverbal information than the TD participants.

**Table 2:** Between-group and within-group fMRI results.

		MNI coordinate				
Right/Left	Anatomical label	x	y	z	cluster size	t value
***Within-group analysis***						
*(Bnv > Bv) in TD*						
Right	AI	32	24	2	271	4.2
Left	AI	−26	22	−2	362	4.5
Right	IFG	50	28	22	154	3.5
Left	IFG	−54	18	20	377	4.1
Left	dmPFC	−4	20	50	201	3.8
Right	SPL	40	−54	56	144	3.9
Left	SPL	−30	−64	48	421	4.2
Left	IPL	−44	−44	52	387	5.5
*(Bnv > Bv) in ASD*						
Left	Amygdala	−26	−4	−18	251	4.1
Right	STP	60	4	0	728	4.2
***Between-group analysis***						
*(Bnv > Bv) × (TD > ASD)*						
Left	ACC/vmPFC	2	34	8	283	4.2
Left	dmPFC	0	30	52	325	4.3
Right	IFG	54	28	22	201	3.6
Right	AI	38	42	−4	148	4.3
Left	AI	−38	16	−8	710	3.5
*(Bnv > Bv) × (ASD > TD)*						
Left	Amygdala	−26	−6	−20	114	4.1
Right	STP	56	2	0	253	4.3

ACC: Anterior cingulate cortex; vmPFC: ventral medial prefrontal cortex; dmPFC: dorsal medial prefrontal cortex; IFG: inferior frontal gyrus; AI: anterior insula; STP: superior temporal pole. P<0.05 FDR-corrected.

### Between-group Analysis

To quantitatively elucidate the differences in brain activity between the ASD and TD subjects, we next conducted a repeated-measure mixed-design two-way ANOVA of the per-voxel brain activity (types of judgments: nonverbal- and verbal-cue-biased judgments × group: ASD and TD). Voxel-level brain activity was extracted from the anatomical brain regions using the same procedures as the within-group analysis. Although there were no significant main effects, we found a significant interaction between the group and the judgment types in the anterior cingulate cortex/ventral mPFC (ACC/vmPFC), dorsal mPFC (dmPFC), bilateral AI, right IFG, left amygdala, and right superior temporal pole (STP) (F(1,30) >12, P<0.05, FDR-corrected). In contrast, the exploratory whole brain analysis did not find any significant activation (P<0.05, FDR-corrected).

A post-hoc analysis of the interaction defined as (Bnv > Bv) × (TD > ASD) found that the ACC/vmPFC, dmPFC, bilateral AIs, and right IFG showed significantly greater activity in the TD group than in the ASD group (t(30) >3.5, P<0.05, FDR-corrected; Fig. 2B and 2C, Table 2). Another post-hoc analysis using the interaction defined as (Bnv > Bv) × (ASD > TD), the left amygdala and right STP showed significantly greater activity in the ASD group than in the TD group (Fig. 2B and 2C, Table 2). These results suggest that brain activity in these regions underlies the impaired use of nonverbal information in ASD individuals.

### Characterization of the Detected Brain Regions

**Figure 3:**
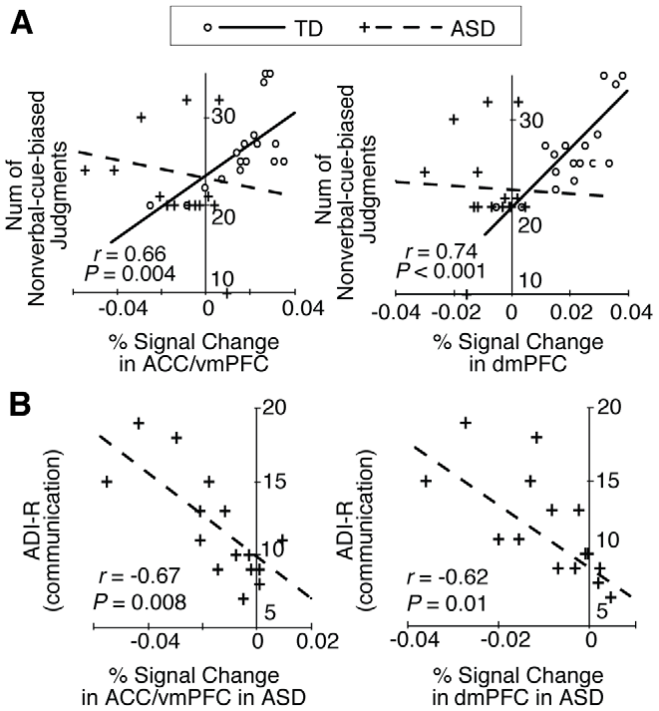
Comparison of fMRI signal and behavioral/clinical indices. **(A) Correlation between fMRI signals and the number of nonverbal-cue-biased judgments.** In the TD group, among the four regions in shown Fig. 2B, only the activity in the ACC/vmPFC and dmPFC showed a significant positive correlation with the number of nonverbal-cue-biased judgments (P<0.05, Bonferroni-corrected). In the ASD group, there was no significant correlation between this behavioral metric and activity in either region. Circles and lines: data of TD participants; + and dashed lines: data of ASD participants. **(B) Correlation between fMRI signal and clinical metrics.** In ASD group, the fMRI signals in the ACC/vmPFC and dmPFC exhibited significant negative correlations with ADI-R communication sub-scores.

We then investigated which of the 8 brain regions examined exhibited activity that was related to the nonverbal-cue-biased judgments by correlating brain activity with the number of the nonverbal-cue-biased judgments. Consequently, in the TD group, activity in only the ACC/vmPFC and dmPFC exhibited significant positive correlations with the number of nonverbal-cue-biased judgments (P<0.05, Bonferroni-corrected among the eight regions in Fig. 2B and 2C; Fig. 3A). In contrast, significant correlations were not found in any of these eight brain regions in the ASD group. In particular, the correlations in the ACC/vmPFC and dmPFC were significantly steeper in the TD than in the ASD group (ACC/vmPFC: Z = 3.3, P = 0.001; dmPFC; Z = 2.9, P = 0.002; Fig. 3A). These results suggest that these two regions are primarily responsible for nonverbal-cue-biased judgments and imply that that decreased activity in these regions may underlie impaired nonverbal-cue-biased judgments in ASD individuals.

### Correlation between fMRI Signals and Symptom Severity

To further examine the relationship between impaired nonverbal-cue-biased judgments and brain activity, we investigated the influence of diminished activity in the ACC/vmPFC and dmPFC on the severity of autistic symptoms related to social interaction (i.e., ADI-R social and communication scores). In both regions, the brain activity significantly and negatively correlated with ADI-R communication scores (ACC/vmPFC: r = −0.67, P = 0.008; dmPFC: r = −0.62, P = 0.01; both were equivalent to P<0.05, Bonferroni-corrected among the two regions and two scores; Fig. 3B), whereas there was no significant correlation between brain activity in these regions and ADI-R social scores (ACC/vmPFC: r = −0.31, P = 0.26; d mPFC: r = −0.21, P = 0.44). These results support the hypothesis that autistic deficits in communication are partially due to diminished brain activity in the ACC/vmPFC and dmPFC.

### Differences in Amygdala Activity

The possibility that ASD group exhibited less overall brain activity than the TD group was excluded by the finding that the ASD group exhibited significantly greater activity in the amygdala than the TD group during responses to congruent emotional stimuli ([36, −2, −28], t = 3.9, P<0.05, FDR-corrected in the amygdala) as well as during nonverbal-cue-biased judgments (Fig. 2D, Table 2).

## Discussion

To the best of our knowledge, the present study is the first to elucidate the neural mechanisms underlying impaired social judgments of incongruent verbal-nonverbal information in ASD individuals. We found that ASD participants showed diminished activity in the bilateral AI, pIFG, ACC/vmPFC and dmPFC. Among these five brain regions, the ACC/vmPFC and dmPFC were the regions primarily involved in nonverbal-cue-biased judgments in the TD participants. In addition, the reduction in brain activity in these regions was predictive of the severity of the ASD participants’ communication deficits. These results indicate that impaired recruitment of the ACC/vmPFC and dmPFC in ASD individuals is associated with their impairments in the social judgment of incongruent verbal-nonverbal information.

The current study employed short movies where a trained professional actor spoke an emotional word while displaying an emotional facial expression and voice prosody. These realistic video stimuli were chosen to maximize the similarity of the psychological task to real-life social situations. In contrast, previous studies have used nonverbal stimuli in the form of a narrator [4], [5], static pictures [55], [56] or cartoons [20]. The current paradigm allowed us to examine the neural basis of more spontaneous social judgments, which is considered to be distinctively impaired in ASD individuals [19]. It is the case that the current short movies may still evoke some unnatural feelings in the participants due to its monochrome style and possible incongruity between disgustful facial expression and contents of some negative words that are unrelated to disgust, and future study is expected to control such incongruity.

Although the present psychological task was a reaction time task, it had no correct response and allowed the participants to express what they felt in a relatively unrestrained manner. Therefore, the present task allowed the participants to respond in a more spontaneous manner than forced-choice tasks. This spontaneity not only reduced the differences between the task and real-life situations, but also enabled us to detect autistic behavioral and neural impairments, which are often difficult to capture in adult individuals with high functioning ASD [57]. ASD individuals with higher verbal abilities can often solve social tasks appropriately when they are given enough verbal information and time to respond [58]–[61]. This superficial, adequate behavior in the adult high functioning ASD individuals is based on ASD-specific forms of the “theory of mind” [58]. In contrast, even individuals with high functioning ASD show difficulties in psychological tasks that require spontaneous mentalizing [3], [18], [19],[62],[63]. In addition, ASD individuals are more likely to exhibit impaired social judgments when psychological tasks utilize nonverbal stimuli [19]. Therefore, in the present study, the task paradigm of allowing the participants to respond more spontaneously than forced-choice tasks may have increased the sensitivity of the task to behavioral and neural impairments in high-functioning ASD individuals.

The number of participants in the present study is comparable to that in previous studies [64], [65], and was large enough to detect a neural basis for impaired social judgments in ASD individuals. Based on the effect size of the detected significant differences in brain activity between the ASD and TD groups (Cohen’s d >1.28), the powers of the present findings were sufficient, even for the weakest activation (>0.93 for the activation in the left AI, t = 3.5; Table 2). Furthermore, the differences between the groups in the present study in terms of sex, age, race, intellectual ability, pharmacological status, and psychiatric comorbidity were at least as small as in previous studies employing ASD individuals [5], [46], [64], [65]. As such, the group sizes and homogeneity were sufficient to support our findings.

By demonstrating significant contributions of the ACC/vmPFC and dmPFC in nonverbal-information-biased judgments, the present results suggest that both areas play important roles in the pathophysiology of communication in ASD individuals. Previous studies have suggested that the vmPFC is involved in self-referential mentalizing [46], [66], [67], and that the dmPFC is associated with non-self-referential mentalizing and deeper objective reasoning [68]–[70]. That is, both of these subregions of the mPFC are involved in mentalizing [23], and impairments of their activity may disrupt social judgments of hostility. The presently reported results are consistent with those previous findings, and suggest more specific functions of these regions during the processing of incongruent verbal-nonverbal social stimuli. Furthermore, this specific role for these brain regions (i.e., social judgments based on nonverbal information) is in accord with previous findings that explicit attention to nonverbal information during an irony comprehension task increased activity in the anterior portion of the dorsal mPFC in ASD children [20].

The right IFG and bilateral AI also exhibited significantly reduced activity during nonverbal-information-biased judgments in the ASD group compared with the TD group. Previous studies in TD individuals have repeatedly reported significant contributions of these brain regions to empathic processing of the emotions and sensations of other people (reviewed in [25], [71]). Functional and structural deficits in these brain regions in ASD individuals have been reported to be associated with clinical deficits in understanding other’s emotional states and in communicating with others [72]–[74]. Notably, consistent with the current results, previous studies have also highlighted a crucial role of the AI in judging and categorizing uncertainty [75].

In contrast to the mPFC, the amygdala showed larger activity in the ASD group than in the TD group. This enhanced activation was observed both during nonverbal-cue-biased judgments and during responding to the congruent stimuli. Anatomical and functional abnormalities in the amygdala of ASD individuals have been reported in a series of previous studies [55], [57], [76], [77]. In particular, previous fMRI studies have suggested that ASD participants show greater activity in the amygdala than TD individuals when they see emotional facial expression [55], [56]. This finding is consistent with the present observation of enhanced amygdala activation during responding to congruent stimuli. Moreover, the present study provided further evidence to support this observation because the enhanced activation in the ASD subjects during the nonverbal-cue-biased judgments could be interpreted as a consequence of an enhanced response to nonverbal information, such as facial expressions.

To validate our findings, we excluded the possibility that all brain regions in the ASD subjects exhibited less brain activity than those in the TD subjects. Even after global normalization, we detected enhanced activation in the amygdala in response to congruent emotional cues in the ASD subjects. This enhanced activation in the amygdala furthermore excludes the possibility that training-related effects influenced our findings. In the present study, before the fMRI scanning, the ASD participants required a longer training period to learn the task than the TD participants (ASD: nine times; TD: three times). If the ASD participants became more habituated to the emotional stimuli during training than the TD controls, they may have exhibited less activity in the amygdala [47]. The enhanced activation in the amygdala in the ASD subjects minimizes this possibility and further validates our main findings.

The behavioral pattern in the response times to incongruent stimuli data excluded the possibility that the ASD participants may have been unable to perceive nonverbal information. If this was the case, ASD individuals would not have been able to sense the incongruence between nonverbal and verbal information. However, as in the TD participants, the response times to incongruent stimuli were significantly prolonged compared to the response times to congruent stimuli in the ASD participants (Fig. 1C), which is consistent with a previous study [5]. In addition, the response times did not differ between the ASD and TD groups, for either incongruent or congruent stimuli, as was observed in another previous study [4]. This behavioral pattern suggests that the ASD participants were able to perceive the verbal and nonverbal information and sense the incongruence of the stimuli, which is in line with a previous behavioral study of conflicting verbal-nonverbal information in ASD individuals [17].

Finally, by estimating the effects of the interaction among the types of judgments and the groups, we also excluded the possibility that activation in the brain regions in which the ASD subjects exhibited less activity reflected conflict monitoring rather than the effects of impaired nonverbal-cue-biased judgments. The detected activations were based on differences in brain activity during the nonverbal-cue-biased judgments compared to verbal-cue-biased judgments. Therefore, we can assume that the influence of the conflict monitoring on these activations was minimal.

In conclusion, the present study demonstrated that significantly decreased brain activity in the right IFG, bilateral AI, and ventral and dorsal mPFC was associated with impaired social judgments of incongruent verbal-nonverbal information in high-functioning non-medicated adult males with ASD. In particular, the nonverbal-cue-biased judgments recruited the ACC/vmPFC and dmPFC in the TD group, and diminished activity in these two regions in the ASD subjects was predictive of the extent of their impairments in communicating with other people. Through disrupting important cognitive systems such as mentalizing, empathy, and judging uncertainty, dysfunction in these brain regions may play a role in shaping the characteristic tendencies of individuals with ASD to make impaired social judgments.

## Supporting Information

Video S1 (MP4)

Video S2 (MP4)

Video S3 (MP4)

Video S4 Each video is a representative stimulus for each type of stimuli used in the present study. In a video, one of 20 professional actors (10 males and 10 females) appears and speaks an emotional word (positive or negative) with emotional facial expression and prosody (happy: positive or disgust: negative). In VideoS1.mp4 and VideoS3.mp4, the actor speaks “(You look) Distressed” in Japanese with disgust or happy facial expression and prosody, respectively. In VideoS2.mp4 and VideoS4.mp4, he speaks “(You are) Friendly” in Japanese with disgust or happy facial expression and prosody, respectively.
(MP4)
